# Editorial: Physical exercise related to student's academic performance

**DOI:** 10.3389/fpsyg.2023.1220616

**Published:** 2023-08-07

**Authors:** Samuel Honório, Marco Batista, Jorge Santos, Matteo Vandoni

**Affiliations:** ^1^Polytechnic Institute of Castelo Branco, Castelo Branco, Portugal; ^2^Sport, Health and Exercise Research Unit, Polytechnic Institute of Castelo Branco, Castelo Branco, Portugal; ^3^University of Pavia, Pavia, Italy; ^4^Unit of Forensic Medicine and Forensic Sciences, Department of Public Health, Experimental and Forensic Medicine, University of Pavia, Pavia, Italy

**Keywords:** exercise, academic performance, psychological wellbeing, physical activity, children

Exercise and physical movement is often defined as any actions promoted by the main muscles group, that results in several forms of energy consumption and expenditure, higher than the usual resting levels and parameters in active people (Leal et al., 2012). Formal, oriented and supervised sport activities gather an extremely important role, in terms of satisfaction, pleasure and cognitive health, promoting much better levels of social interconnection, developing higher levels of self-esteem and wellbeing. Children and teenagers who are more physically active have more stabilized blood pressure values considered healthy and less tendency to overweight once compared to inactive children (Bois et al., 2005). Identifying conceptual contexts that correctly define the term physical activity becomes difficult, due to the variety of existing concepts and the way in which several authors understand what the practice of this variable effectively. It's often confused with practices whose movement does not result in caloric expenditure or beneficial changes of parameters evaluated as being healthy (Ortega et al., 2008).

The learning process, a factor also associated with physical activity, is intended as the capacity to understand and use properly the information to acquire specific skills and knowledge, that gives students a rich school experience, developing competences to elaborated answers, that helps developing skills taken as acquired, internalized and consolidated (Santos, 2014). Loureiro (2012) states that learning is a process of acquiring information and behavior patterns, which is reflected in the increase in the repertoire of skills and in which the modification of behavior results from practice and experience. Learning constitutes a fundamental element in human development, in which learning implies retaining what is acquired through practice and repetition. For Ferreira (2010), the brain changes when faced with new learning and these must be integrated into prior knowledge to be given significance. As the nerve pathways are created and exercised, learning in turn also develops. Lipscomb (2007) states that when the development processes of thought, language and imagination come together, new representations emerge, which is the trajectory of knowledge construction. The are indicators that, moderate physical activity, increases cognition. Recently conclusions were observed and reported that the brain presents marked cognitive improvements with the practice of physical exercise (Berchtold et al., 2005). Also, according to Silva and Cubas (2009) physical efforts provide several benefits directly to the nervous system inducing the production of neurologic features. The practice of physical activity enhances the production of glutamatergic neurons on the neurologic synapses, that help and are responsible for the cognitive and motor functions of the nervous center when performing exercise. According to Vaynman (2005) exercise can develop the activation of neurological circuits that can change the perception of the transmitted information by synapses, activating specific molecules that benefit the brain function for motor achievement. Regular physical activity is considered a factor that across the synapse, the transmitting neuron called the pre-synaptic cell, while the receptor called the post-synaptic cell, causes nerve cells to send chemical messages with neurotransmitters in one direction across the synapse from the pre-synaptic cell to the post-synaptic improving processing capacity at the motor level (Lam and Riba, 2016). Physical activity brings benefits that are related to better achievements in brain function in terms of cognition, mentality and reasoning, inducing positive behaviors of wellbeing avoiding symptoms of depression, thus, facilitation the process of learning (Berchtold et al., 2005).

All these characteristics are directly interconnected with academic performance, turning a very interesting topic, because it has influence in many fields and contexts of children's lives. When these contexts are experienced in a wrong way, greater would be the possibility of higher incompetence (Rosa, 2016). Academic performance is intended to be a way to assess children's knowledge about certain issues, in this case on a school context. However, it is no more than registering a qualitative or quantitative value on students' grades resulting from the schoolwork they do. These results are also disclosed for students, parents and teachers that translates the student capacity obtained (Paz, 2014). The function of evaluation, according to Desai et al. (2015), is to evaluate and certify, measure and verify the degree of fulfillment of objectives. It can also be added that the objective of this evaluation is to summarize the performance of the students, in a group of strategies and learning objectives and that it was designed so that it is possible to make decisions about the results of the evaluated ones. It is with this type of evaluation that teachers classify and send their results and necessary information to parents and students (Martins, 2012). Academic performance is then the externally evaluated result achieved by the student. Several variables have been associated with school results, that is, academic performance, such as: self-esteem (representation of oneself under an affective component) and self-concept (beliefs about oneself from a cognitive perspective), parents' schooling as well as their involvement in their children's lives, and the sociocultural context in which the child or adolescent is inserted (Oliveira, 2015). So, it is easily noticeable that, exercise and/or sports activities are directly connected to develop positively academic performance, cognitive function, self-confidence, socialization, memory, self-perception of body image and wellbeing (Rigoli et al., 2012).

The articles published in this Research Topic frame and support the contexts highlighted according to the themes under study.

## Author contributions

All authors listed have made a substantial, direct, and intellectual contribution to the work and approved it for publication.
